# Educating patient-centered, systems-aware physicians: a qualitative analysis of medical student perceptions of value-added clinical systems learning roles

**DOI:** 10.1186/s12909-018-1345-5

**Published:** 2018-11-01

**Authors:** Jed D. Gonzalo, Daniel Wolpaw, Deanna Graaf, Britta M. Thompson

**Affiliations:** 1 Medicine and Public Health Sciences and Associate Dean for Health Systems Education, Penn State College of Medicine, Hershey, PA USA; 2 Medicine and Humanities, Senior Consultant for Educational Innovation at the Regional Medical Campus, Penn State College of Medicine, Hershey, PA USA; 3 Office of Medical Education, Penn State College of Medicine, Hershey, PA USA; 4 Medicine and Associate Dean for Learner Assessment and Program Evaluation, Penn State College of Medicine, Hershey, PA USA; 5 Division of General Internal Medicine, Penn State Hershey Medical Center – HO34, 500 University Drive Hershey, Hershey, PA 17033 USA

**Keywords:** Undergraduate medical education, Value-added medical education, Health systems science, Experiential learning, Health systems, Social determinants of health, Patient-centered care

## Abstract

**Background:**

Medical schools have a critical need to develop roles for students that are “value-added,” defined as “…*experiential roles that can positively impact health outcomes while also enhancing student knowledge, attitudes, and skills in Clinical or Health Systems Science*.” Following implementation of value-added clinical systems learning roles for all first-year students, authors investigated student perceptions of the educational value from these patient-centered experiences.

**Methods:**

Between 2014 and 16, authors collected logs from students following their working with patients; authors also performed six, 1:1 student interviews, which were audio recorded and transcribed verbatim. Authors used thematic analysis to explore students’ perceptions of the experience and educational benefits from these roles. Authors identified themes, and agreed upon results and quotations.

**Results:**

A total of 792 logs from 363 patients and six interviews were completed and analyzed. Students reported six educational benefits of performing value-added clinical systems learning roles in the health system, including enhanced understanding of and appreciation for a patient’s perspective on health care and his/her health, barriers and social determinants of health, health care systems and delivery, interprofessional collaboration and teamwork, clinical medicine, and approach to communicating with patients.

**Conclusions:**

Students’ reported educational benefits from value-added clinical systems learning roles span several learning areas that align with clinical and Health Systems Science, i.e. the needs of future physicians. These roles have the potential to shift learning from the physician-centric identity to one more fully aligned with patient-centered, team-based providers, while also potentially improving health today.

**Electronic supplementary material:**

The online version of this article (10.1186/s12909-018-1345-5) contains supplementary material, which is available to authorized users.

## Background

Transformational changes in health care delivery and evolving perspectives on physician roles are precipitating a major re-visioning of medical education. Health Systems Science (HSS), including concepts of population and public health, high-value care, and quality improvement, can no longer be relegated to the “other” file of the curriculum, and demand meaningful integration with traditional basic and clinical sciences [1–5]. At the same time, renewed focus on the importance of experiential and workplace learning, long a staple of clinical education that has arguably been diminished in the current supervisory and economic environment, has opened up the critical question of how medical students might “add value” as they pursue learning in HSS [6–10]. Social learning theory, specifically the concepts of legitimate peripheral participation and communities of practice advanced by Lave and Wenger posits that progressively increasing authentic participation is a transformational process in the socialization and professional identity formation of learners in medicine [11–13]. While student participation in traditional clinical rotations focuses on developing clinical skills (i.e., history taking, physical examination), value-added clinical systems learning roles are designed to provide students with opportunities to engage in HSS and clinical skills while adding value to the health care system by legitimately contributing to patient care [11, 12, 14].

Despite recommendations to develop value-added opportunities for medical students, early experiential programs still primarily focus on clinical sciences and preceptorships [15]. While potentially beneficial for students, these roles are generally considered a burden for preceptors and practices, and the benefit to the patient is not clear [16, 17]. In contrast, value-added clinical systems learning roles have been proposed as a primary method to not only develop students’ knowledge and skills in areas such as HSS, but also make contributions to care delivery [7–9]. Our preliminary work on value-added clinical systems learning roles have identified the need for these roles to provide an educational experience that aligns with the evolving needs for future physicians, and are not “service-heavy, education-lite” experiences [18].

Examples of value-added clinical systems learning roles are limited within the literature, and include pilot programs in patient navigation and health coaching [19, 20]. While in theory these roles might provide value to the health care system, there are no studies that investigate their educational value. In this study, we explored this issue from the student perspective based on their year-long experiences as patient navigators. Our primary research question was: What do students experience as educationally valuable by participating in value-added clinical systems learning roles?

## Methods

### Study setting

In academic year 2014–2015, Penn State College of Medicine implemented a Systems Navigation Curriculum, with the primary goals of aligning medical education with health system needs and advancing students’ competence in HSS [2, 21]. The new curriculum included two components: (1) a health systems course (> 100 contact hours) in the first year focusing on topics such as insurance, cost, care coordination, population and public health, social determinants of health, high-value care, teamwork, and leadership, and, (2) an authentic clinical systems learning role as a patient navigator [2]. The patient navigator role is distinctly different from traditional clinical or service-learning rotations, and fulfills several criteria: 1) provides an authentic clinical community of practice experience, 2) allows for direct experience with core HSS concepts, and, 3) creates the opportunity for students to view the system from the perspective of the patient [22]. Patient navigation has historically used outreach workers to explore patients’ barriers to care and help patients navigate complex health care systems to obtain required care and reduce disparities [23]. Although founded and primarily used in oncology, we have broadened the concept to include a broad range of tasks, including health coaching, transition planning and implementation, and patient education. These tasks were identified by clinical care teams as necessary to improve care at their sites, and involved face-to-face meetings, telephone calls, and/or home visits [24, 25]. Following an orientation (10 h), students were integrated into interprofessional care teams in one clinical site or program (17 sites in year 1, 36 sites in year 2). Mentors were identified within each site for the students. Students participated in these sites two to three afternoons per month between September and May. The 2014–15 academic year included 85 of 150 students (17 sites were sufficiently developed to integrate 85 students), while the 2015–16 year included 144 of 150 students. The types of clinical sites included: (1) primary care clinics, (2) specialty-based clinics, (3) underserved/free clinics, and (4) transitions programs (only included in the 2015–16 year).

### Study approach

Since limited literature existed related to the educational benefit of value-added clinical systems learning roles, we used an inductive approach. We used constant comparative methods to analyze our data, and triangulated our data via two primary sources: (1) students’ monthly logs of patient encounters, and, (2) in-depth, semi-structured interviews with six students following 1 year as patient navigators. The six interviews were conducted to triangulate data collection as well as capture additional individual-level data. The study was exempt from further review by the Institutional Review Board at the Penn State College of Medicine as an educational assessment activity.

### Data sources and collection

First, as part of ongoing educational assessment, students completed an online log at the end of each patient experience or each month (if the experience lasted ≥1 month). The log included questions related to activities performed, barriers encountered by patients, and the educational value from the experience (Additional file 1). Second, in-depth semi-structured interviews were conducted with students from a diverse range of clinical sites. Invitations were sent via email to a sampling of student patient navigators from across the three clinical site types involved in the first year of the program; the first six to respond were scheduled for an interview. Questions regarding student’s experiences and the potential educational value, if any, from performing assigned tasks as a patient navigator were explored (Additional file 2). A professional transcriptionist transcribed each recording verbatim.Data Analysis.

Three members of the research team experienced in qualitative research analyzed the data [3, 26]. At the beginning of our analysis, our research team acknowledged our own biases related to the potential value of patient navigator roles and HSS. Therefore, we used caution when analyzing logs so as to not over-interpret them, and asked neutral questions during the one-on-one interviews. We additionally performed several cross checks during data analysis to ensure confirmability of our results [27].

We approached our data using the constant comparative method [28]. Two investigators (J.G., D.G.) independently analyzed a small sample of the 2014–15 logs (*n* = 20), and identified initial themes and categories to generate a preliminary codebook, with four adjudication sessions [17–19]. These two investigators discussed categories and themes, and created a codebook. Two investigators (D.G., B.T.) then independently analyzed all data (all submitted logs and interview transcripts), with several additional adjudication sessions, during which initial categories and themes were compared for consistency and agreement, and disagreements were discussed until agreements were reached. When disagreements occurred, consensus was reached through discussion. The research team had frequent meetings to discuss disagreements, collapse and modify codes as necessary, and extract themes from the developed codes, enhancing credibility of this work [27]. All investigators agreed upon final themes and selected quotations. We used data management support program NVivo 10 QSR International.

## Results

Between September 2014 and December 2016, a total of 792 logs were collected from first-year medical students (*n* = 307 in year 1, *n* = 485 in year 2), related to at least 363 unique patients (students may have submitted several logs on the same patient). Responses ranged from one sentence to several pages in length. In May 2015, a total of six interviews were completed (range 36–71 min); three students were male and three were female. Our analysis identified six categories of educational value to students who participated in the patient navigator program, as identified below. A total of 1,268 coding references was applied to the data in the analysis – the number and percentage of the total of coding references are also included below.

### Patient barriers to health (*n* = 379, 30%)

Students reported learning about barriers to care in a direct way, including insurance, disability, and social determinants of health and factors that augmented/prevented receipt of health for patients. This theme was directly related to the health issues encountered by patients.

- *Working with [Patient A] to figure out how to best fill his prescriptions and navigate his new work environment were both novel experiences for me. While it seems like a simple matter to fill medications, [Patient A’s] financial situation and distance from a suitable pharmacy made this task quite difficult. It took a fair amount of coaching to get [Patient A] in touch with his PCP and get him the medications he needs.*
- *This experience has helped me understand the difficulties and barriers certain groups of people, like the homeless, experience that are often taken for granted by more well-off people. Scheduling a doctor's appointment for a physical can be super complicated by insurances, or lack thereof, as well as transportation and ability to pay copays if necessary. It has greatly changed my perspective of how to navigate healthcare from different standpoints and made me want to be a more socially-conscious physician.*
- *While the patient had some clear medical issues, his main complaint was outside of medicine. We therefore had to access resources in a field we were not well versed on. It exemplified how issues outside of medicine, like being unemployed and unable to even take the high school equivalency test to find employment, can have an effect on a person’s health.*
- *I learned how strokes occur, since he was my first patient. I also learned the consequences of having a stroke, and how it can change a person's life completely. This man lost his job, cannot drive himself around, and has to take care of himself with no family support. With his memory failing him, taking care of himself and making it to all of his appointments cannot be easy. He is making significant lifestyle changes.*

### Patient’s perspective on health care and his/her health (*n* = 344, 27%)

Students identified the benefit of learning about issues related to a patient’s life, including an enhanced awareness and understanding of the patient perspective in his/her environment. This theme specifically highlighted the opportunity for students to develop meaningful relationships with patients at early stages of their medical careers.

- *You get to know so much more about [the patients] than just talking over the phone. You can be talking on the phone and they sound like they have it all together. You get to their home and it’s chaos. There are medications in every room. You get a good picture of what their life is really like.*
- *There was an individual who had some mental health difficulties that lived on his own. He had no family in the area. It definitely made me appreciate the question: “Do you have any spiritual or family support?” When I learned more about him, he literally goes home to a one bedroom he’s renting, in a house full of others who are just renting one bedroom. They cook together in a big kitchen but people alternate who cooks for any given night, so no one really knows how to cook his low-sodium meal. He doesn’t have the ability to express that. He understands he’s not supposed to eat salt but he couldn’t teach somebody else that he’s not supposed to eat salt. It would never happen based on his mental difficulties.*
- *I’m seeing the other side of medicine. I see patients who come to the clinic after their time with the physician. It’s interesting to see how confused they become. I am sure the doctor is explaining the instructions well during their visit, but patients often forget what the doctor said, or had a question develop once they began their routine.*

### Health care system and delivery (*n* = 219, 17%)

Students highlighted components or processes of the larger health care system and how care is delivered, which included policy, informatics, and insurance. This category was related to but distinct from patient barriers in that the identified learning was not related to a specific patient but reflected the larger context of the health care system. Students also frequently identified insufficiencies of the health system that required improvement. For example, students frequently identified the importance of transportation to access care and the availability and access to insurance for patients cared for by providers in clinical locations.

- *I am getting a real hands-on experience getting to know the health care system and how it affects the care of certain patients. You really get a real understanding of the troubles and frustrations it causes patients. It makes me more aware as a future physician of problems many of my patients may face and pushes me to have more of a role in change.*
- *[This] was an interesting example of problems with the health care system. Even though patients were previously approved for discharge, their discharges were being withheld because of a technical failure. This will ultimately cost the patient and hospital a lot of money.*
- *I learned a lot about what patients must deal with trying to establish care in our complicated health system. I learned about the different services available for patients living in poverty and with disabilities and how complicated and time consuming it can be to obtain services. I was able to work through the process of applying for medical assistance and working with a hospital system to receive financial aid to cover thousands of dollars of unpaid bills that were left over from Medicare coverage. It is not as easy as filling out a form and getting coverage, and it often takes multiple follow-up calls to state agencies just for someone to look at your application.*

### Communicating with patients (*n* = 136, 11%)

Students identified benefits to learning how to communicate with patients about their medical problems or follow-up with their primary care physicians, or with other providers. Students reflected upon the learning process and how they might change their approach to similar situations in the future.

- *He helped me learn my approach should have been a bit more firm. I gave him time, allowed him to call the health department back and set up his own appointment. I should have set up an appointment for him and said, “all right, we’re expecting you here.” He wasn’t really on board. He got discharged from the care because we couldn’t follow up. I’ve thought about how I approached the situation and ways I could of had him finish.*
- *This [patient] was very willing to work with me, despite his insistent desire to go home and not be in the facility. I see the importance of one accepting when assistance is needed, and this further strengthened my ability to counsel a patient on this topic.*
- *Being handed real patients and trying to help them figure out their complicated problems is a very real-life gritty experience. You learn immediately how to speak with people that are having hard times with health and the system. You learn how to react, respond, and you try to learn what you can do to help.*

### Interprofessional collaboration and teamwork (*n* = 133, 11%)

Throughout their role as a patient navigator, students reported learning about the roles and responsibilities of other health care providers and collaborating with them to help patients they were navigating achieve optimal outcomes.

- *The [physician] knew everything about tuberculosis, how it interacted in societies, how it would shape a patient’s life and things they might encounter. The [nurse coordinator] knows what needs to be done for these patients. They need a chest x-ray, they need a follow-up here and the kind of family dynamics, and here are the problems we’re having.*
- *I appreciated how the nursing staff was able to get him to understand his disease. As a medical student taking cardiology, I understood heart failure in a different language. But what they explained to the patient was sufficient for them to truly grasp what it was that was going on within their body and helped him understand why the changes that he was going to make would impact his health.*
- *It takes a great deal of patience to do the job social workers do. Working under a social worker, I was consistently impressed by her knowledge of the health system at both the hospital and government level, and her patience when working with patients who did not always have the greatest sense of personal responsibility. Doing some of the [social worker] tasks, I have a better understanding of a social worker's vital role on a medical team. In the future I will have a better understanding of the ways in which they assist patients and know when to refer patients to them.*

### Clinical medicine (*n* = 57, 4%)

Although placed in experiential roles with the primary goal of education in HSS, students frequently identified the clinical educational benefit of learning about diseases, diagnoses, surgeries, and therapeutics in the course of helping to navigate their patients.

- *I have learned a lot about ALS as a disease, witnessing patients at various stages of cognitive and physical function. Additionally, I experienced how external factors such as family, faith and community support can play into the willingness of patients to comply to clinic recommendations and quality of life. ALS is not an easy disease to navigate- the effects it can have emotionally, physically and mentally are difficult to witness.*
- *I have far more insight into the transplant process. I saw some of the paperwork (quite extensive), discussed some of the lab tests that needed to be done, and gained insight into patient education the hospital provides.*

## Discussion

In this study, we explored first-year medical student learning experiences associated with value-added clinical systems learning roles with the goal of advancing Health Systems Science (HSS) education in medical schools. Our data suggest value-added clinical systems learning roles can provide students with learning opportunities while potentially adding value to the system, challenging the assumption that value-added work is primarily “service work” and void of educational benefit. As transformational changes occur in health care delivery, medical education is beginning to address the need for the integration of Health Systems Science (HSS), including concepts of population and public health, high-value care, and quality improvement, with the traditional basic and clinical sciences [1–5]. These advances in the understanding and configuration of HSS have created the expectation of better educational alignment and a clear need for these new roles [3]. Although an increasing number of educators and medical schools have advocated for value-added roles, little work has identified educational benefit from the student perspective [2, 7, 9, 29, 30].

Our study supports the concept that value-added clinical systems learning roles have potential to enhance student learning about patients’ barriers to care and their perspectives in care delivery (e.g. barriers to optimal health), HSS (e.g. insurance, policy, care delivery, and teamwork and collaboration), and clinical and communication skills [3, 31]. In addition, students had the opportunity to see beyond traditional physician-centric perspectives, engage in the lived experience of their patients, and participate as authentic members of health care teams. This aligns well with evolving health care organizations that increasingly regard HSS and team education as essential physician skills [4, 32–36]. While clarifying some aspects of the value-added role concept, these results also create important new questions, such as “How much and what type of clinical science and communication skills can these roles contribute to early medical student education?”

In addition to learning HSS and clinical medicine concepts, the patient navigator experiences provided opportunities for students to work with non-physician health care professionals to make meaningful contributions to the care of patients and populations. Students were able to work as members of interprofessional care teams and gain deeper insights into each member’s role and expertise. It is important to note that these insights are not limited to knowledge of other professions – they are based on authentic working relationships that allow students to experience firsthand how they, and the physicians they aspire to be, actually function on an interprofessional team. This educational exposure is unlike much of current-day interprofessional education that occurs in simulated environments, usually on a short-term basis [37]. Traditional physician-centric clinical experiences (preceptorships), even when augmented by designed interprofessional activities, may not be enough to educate students for effective practice in evolving systems of care. Our data suggest that embedding students onto interprofessional care teams in authentic non-physician roles can help shape student interprofessional identity and abilities [33].

These results also speak to the need to “push” experiential learning beyond the doctor-patient office or bedside classroom, targeting collaborative care teams that focus on areas such as social determinants of health, population health management, and patients’ experiences with insufficiencies in health systems. Clinical preceptorships have generally not focused on these areas of learning [38]. As reported by our students, value-added clinical systems learning roles can allow for authentic contributions to health care teams early in training, creating opportunities for students to be integral members of the team rather than “fifth wheels” in patient care. In the current clinical practice environment, students in traditional physician-centric pathway often languish outside of a legitimate peripheral participation role, significantly delaying their entry into a meaningful community of practice. In contrast, value-added clinical systems learning roles focus on lower-stakes tasks, and in this study, students reported authentic engagement in the clinical setting with associated learning opportunities. We believe this is a critically important area of attention and investment in medical education. The student voices in this study are one of the first early indications that medical students can potentially make a difference on interprofessional care teams, learning from other professionals as they work in authentic roles.

This study has several limitations. First, students self-selected to participate in the interviews, raising the possibility of selection bias. However, because we triangulated our data through use of both interviews and patient logs, we believe these results have credibility, and are applicable to other settings where value-added roles are being implemented. Students were assigned to one clinical site, each with its own unique characteristics (e.g. clinical processes, patient populations), which may limit any one student’s experience of the breadth of themes identified in this study. Several investigators in this project were also the same individuals leading and facilitating the educational experience, which raises the possibility of bias in the analysis phase; we sought to address this concern by using multiple coders and cross checks by the larger research team. Next, this work represents early evaluation of the program, therefore we do not have data regarding the impact on these students’ future behavior or practice [39]. Last, since our results included only one school, our context may not be applicable to other schools, thereby limiting the transferability of these findings [27]. However, experiences captured in the logs and interviews were from 36 different clinical sites and settings, enhancing the diversity of these results.

## Conclusion

In conclusion, we analyzed logs and interview transcripts from first-year medical students who had been performing meaningful clinical systems learning roles. We identified several educational benefits including learning in social determinants of health, patient’s perspectives of their health issues, interprofessional collaboration, clinical learning, and communication skills. These results demonstrate the educational potential for providing students with value-added experiential opportunities to learn Health Systems and clinical sciences. We believe these student experiences have clear potential to be a win-win for health care systems, patients and educational programs. Importantly, this study suggests that authentic non physician-centric roles in the health system can enhance learning; additional research is needed to determine how much value these roles actually provide to patients and health systems.

## Additional file

Additional file 1: **Appendix 1.** Patient Navigator Log Questions. (DOCX 19 kb)

Additional file 2: **Appendix 2.** In-Depth Interview Protocol. (DOCX 19 kb)

## Abbreviations

HSS: Health systems science
PCP: Primary care physician
