# Supplementary material for: Educating patient-centered, systems-aware physicians: a qualitative analysis of medical student perceptions of value-added clinical systems learning roles
Source: BMC Med Educ. 2018 Nov 1;18:248. doi: 10.1186/s12909-018-1345-5 (PMC6211412; doi:10.1186/s12909-018-1345-5)
Supplement: Supplementary file 1 — Appendix 1. Patient Navigator Log Questions. (DOCX 19 kb) [file 12909_2018_1345_MOESM1_ESM.docx]

**Appendices:**

**Appendix 1:**

**Patient Navigator Log Questions**

1. Student name: __________________________________
2. Please identify your patient navigation site:

| Bethesda Mission | HMC Skin Oncology | Lebanon Valley Free Clinic |
| --- | --- | --- |
| Holy Spirit Health System | HMC Rehab | Lebanon Volunteers in Medicine |
| HMC Breast Clinic | HMC Renal Dialysis | Lebanon VA Medical Center |
| HMC Stroke Program | HMC Emergency Department | Pennsylvania Psychiatric Institute |
| HMC Fishburn Family Medicine | HMC Pre-Anesthesia | Pinnacle Community |
| HMC Nyes Road I | HMC Pain Management | Pinnacle Kline |
| HMC Nyes Road II | HMC General Internal Medicine | Pinnacle Medical Group |
| HMC Harrisburg Family Medicine | HMC 6th Floor Acute Care | Pinnacle REACCH |
| HMC Inflammatory Bowel Disease Clinic | HMC Surgical Weight Loss | Pinnacle Spine |
| HMC Infectious Disease | HMC Internal Medicine East Campus | Pinnacle Stroke |
| HMC Emergency Department | HMC Neurology/ALS Clinic | Pinnacle Surgery |
| HMC Pediatric Hematology | Lancaster General Care Connections |  |

1. Date you started navigating the patient: __________________________________
2. Patient's MRN: __________________________________
3. Patient's Date of Birth: __________________________________
4. Think about the number of times that you met or worked with or on behalf this patient during the past month. Please provide a numerical value for the questions below:
   1. Number of home visits (if any):
   2. Number of face-to-face meeting in-hospital or clinic setting:
   3. Number of phone conversations with the patient or family:
   4. Number of phone conversations ON BEHALF of the patient (e.g. with providers or individuals other than the patient or family):
5. Please provide a detailed response to the questions below:
   1. Regarding this patient, why was this patient initially identified for patient navigation? Please provide the

patient's backstory, and the barriers at play in this patient's care: __________________________________

- 1. Please describe how you played a role as a navigator in this case (e.g. connected the patient to resources,

provided emotional support, etc.): __________________________________

- 1. To date, have you seen any change in the patient's behavior (e.g. lower readmission rates, attitude, etc.) since

you started navigating him or her? __________________________________

- 1. Were there any issues you encountered with this patient that you were unable to address or handle? Yes No
  2. Please describe the issue(s) and WHO you contacted for assistance with the issue(s):

__________________________________

- 1. What are your next steps in continuing to navigate this patient? __________________________________
  2. Regarding this case, could you propose any potential improvements in the health care system? Think

broadly; it could include the hospital/clinic setting, community, or patient's home that may improve this

patient's health or other patients in a similar situation. __________________________________

- 1. How is the experience educationally valuable to you? __________________________________
