# Supplementary material for: Educating patient-centered, systems-aware physicians: a qualitative analysis of medical student perceptions of value-added clinical systems learning roles
Source: BMC Med Educ. 2018 Nov 1;18:248. doi: 10.1186/s12909-018-1345-5 (PMC6211412; doi:10.1186/s12909-018-1345-5)
Supplement: Supplementary file 2 — Appendix 2. In-Depth Interview Protocol. (DOCX 19 kb) [file 12909_2018_1345_MOESM2_ESM.docx]

**Appendix 2:**

**In-Depth Interview Protocol**

**We are seeking your perspectives about patient navigation within your sites over the past year. In responding to these questions, please consider the context of your patient navigation sites.**

1. Representative open-ended questions:
   1. Based on your role as a navigator, what have you learned about the system?
   2. In which situations during your patient navigation did you find most valuable to your career?
   3. As a future doctor, how do you see your patient navigator experiences advancing your knowledge and skills for your career?
   4. If an incoming first-year student asked you about patient navigation, how would you explain the benefits to the program to them?
   5. Questions about learning as a navigator and learning in classroom.
   6. Give me an example of something that you saw as a navigator that you had learned about in the classroom SyNC sessions?
      1. Probe the story
   7. Tell me a story about something that you saw as a navigator that you subsequently learned more about during one of the SyNC sessions?
   8. Think about a successful situation with a patient during navigation. Please share that example.
      1. What made the encounter successful?
      2. What did you learn from that experience?

Think about an unsuccessful patient navigator encounter that you experienced this year. Please share that example.

- - 1. What made the encounter unsuccessful?
    2. What did you learn from that experience?

1. Describe the relationship between your learning as a navigator and your learning in the SyNC sessions?
   - 1. How, if at all, did they reinforce one another?
     2. How, if at all, did they detract from one another?
     3. If student does not see any connection, probe why they saw no connection between the two.
2. Were there any other positive aspects of patient navigation?
   1. Can you think of any additional benefits?
3. Are there any other stories from your navigation experience that you would like to share?
